# Observing prosociality and talent: the emotional characteristics and behavioral outcomes of elevation and admiration in 6.5- to 8.5-year-old children

**DOI:** 10.3389/fpsyg.2024.1392331

**Published:** 2024-05-24

**Authors:** Sina Gibhardt, Robert Hepach, Annette M. E. Henderson

**Affiliations:** ^1^Faculty of Psychology, University of Auckland, Auckland, New Zealand; ^2^Faculty of Education, Leipzig University, Leipzig, Germany; ^3^Department of Experimental Psychology, Oxford University, Oxford, United Kingdom

**Keywords:** prosocial emotions, elevation, admiration, prosocial development, middle childhood

## Abstract

Helping and seeing others being helped elicits positive emotions in young children but little is known about the nature of these emotions, especially in middle childhood. Here we examined the specific emotional characteristics and behavioral outcomes of two closely related other-praising moral emotions: elevation and admiration. We exposed 182 6.5- to 8.5-year-old children living in New Zealand, to an elevation- and admiration-inducing video clip. Afterwards children’s emotion experiences and prosocial behaviour was measured. Findings revealed higher levels of happiness, care, and warmth after seeing prosociality in others (elevation condition) and higher levels of upliftment after seeing talent in others (admiration condition). We found no differences in prosocial behavior between the elevation and admiration conditions. This is the first study to assess elevation in childhood and offers a novel paradigm to investigate the role of moral emotions as potential motivators underlying helping.

## Introduction

Children’s prosociality is well-documented but the emotional motivators underlying prosociality remain debated (Hepach et al., 2013; Davidov et al., 2016). Moral emotions have been argued to sustain at least three forms of prosocial behavior (i.e., helping, sharing, and cooperation) allowing young children to maintain social relationships by engaging in other-oriented actions even when helping comes at a cost to themselves (Ongley and Malti, 2014; Malti and Dys, 2015; Vaish and Hepach, 2020). In recent years, the moral emotion of elevation has been proposed to be an important motivator of prosocial behavior in adults (Haidt, 2000; Schnall et al., 2010; Lai et al., 2014; Thomson and Siegel, 2017). According to theory and research with adults, elevation is elicited when witnessing moral excellence in others resulting in distinct emotional characteristics and prosocial motivation (Haidt, 2003; Thomson and Siegel, 2013). Specifically, witnessing moral excellence (e.g., prosociality) in others is argued to elicit distinct emotional (e.g., elevation) and motivational outcomes (e.g., helping) compared to witnessing excellence not specific to morality (e.g., talent; admiration where the outcome is a motivation to excel oneself) (Algoe and Haidt, 2009). However, to date, no studies have compared the specific emotional experiences and behavioral outcomes in children of seeing others being helped (moral excellence) versus seeing talent (non-moral excellence). Since prosocial behaviors emerge in early childhood and become increasingly complex and selective across middle childhood (Warneken and Tomasello, 2009; Svetlova et al., 2010; Dunfield, 2014; Hammond et al., 2017; Aitken et al., 2020; Grueneisen and Warneken, 2022; Gibhardt et al., 2024), understanding the role of moral emotions underlying prosocial development is crucial (Vaish and Hepach, 2020).

### The development of moral emotions

Moral emotions, both positively and negatively valenced, have been proposed to be a motivator underlying prosociality in young children (Ongley and Malti, 2014; Vaish and Hepach, 2020). Support for the role of negatively valenced moral emotions supporting cooperation comes from evidence demonstrating that the elicitation of negative moral emotions such as guilt in children as young as 2- to 3-years of age is associated with prosocial motivations, for example, to repair social relationships that have been harmed by broken trust (e.g., Vaish et al., 2016; Vaish, 2018; Vaish and Grossmann, 2022). By contrast, positively valenced moral emotions such as gratitude are evident from around 3-years of age with young children being more motivated to be generous toward individuals who previously benefitted them (e.g., Vaish et al., 2018; Hepach et al., 2019). Together, the findings from prior work support the possibility that moral emotions are an important motivator to maintain, sustain, and repair social relationships and thus may act as a moral compass throughout prosocial development. However, what findings from previous work leave open is whether moral emotions can also be *elicited* by simply observing prosociality in others, without being directly involved. One candidate for such an emotion is *elevation*.

### The other-praising emotions of elevation and admiration

The positive emotion of elevation has been proposed to be a possible motivator underlying adults’ tendency to engage in prosocial behaviors (Landis et al., 2009; Schnall et al., 2010; Thomson and Siegel, 2013). Elevation is a positive, discrete emotion elicited when witnessing acts of moral goodness and kindness (Haidt, 2000, 2003). Haidt described elevation as a sensation of warmth and an uplifting feeling in the chest that makes people seek connections with others. Support for this account comes from findings that elevation is elicited in adults when they witness acts of moral goodness such as viewing a video clip of a person helping another, remembering witnessing another doing a charitable act, or reading a story about a moral exemplar (Haidt, 2003; Silvers and Haidt, 2008; Algoe and Haidt, 2009; Freeman et al., 2009; Cox, 2010; Schnall et al., 2010; Lai et al., 2014; Thomson and Siegel, 2017). Critically, in adults, elevation is a distinctively positive emotion characterized by a feeling of upliftment, warmth in the chest, and optimism about humanity (Haidt, 2000; Silvers and Haidt, 2008; Algoe and Haidt, 2009; Schnall et al., 2010; Oliver et al., 2012; Diessner et al., 2013; Siegel et al., 2014). To date, the evidence suggests that elevation is associated with unique psychological, physiological, and behavioral outcomes that are distinguishable from other positive moral emotions such as amusement (Silvers and Haidt, 2008), awe (Keltner and Haidt, 2003; Schnall et al., 2010), happiness (Oliver et al., 2012), and gratitude (Algoe and Haidt, 2009).

Furthermore, elevation has been proposed to differ from the closely related ‘other-praising’ emotion *admiration* (Diessner et al., 2013). Elevation is argued to be specific to witnessing *moral* excellence such as prosociality (Silvers and Haidt, 2008; Schnall et al., 2010), whereas admiration is elicited when witnessing excellence not directly related to morality such as talent (Algoe and Haidt, 2009; Thomson and Siegel, 2017). Specifically, admiration is a non-ethically relevant emotion and can be defined as a type of surprise that is linked to pleasure and approval (Darwin, 1890/2007; Körner et al., 2016; Malti et al., 2020). Admiration is felt based on ranking of skill or ability, that is, it is elicited when another person exceeds a set standard of achievement (Schindler et al., 2013).

Indeed, an empirical study by Algoe and Haidt (2009) supports the distinction between elevation and admiration by showing that these emotions have different emotional and motivational effects. In the study, the researchers exposed adults to elevation and admiration inducing video clips and assessed participants’ emotional experiences and motivational effects via a self-report measure. Overall, Algoe and Haidt found that inducing elevation led to distinct emotional experiences in adults such as warmth in the chest and relaxed muscles whereas admiration led to high energy and increased heart rate. Furthermore, findings revealed distinct motivational effects such as a desire to be a better person and to do something good for another in the elevation condition compared to a desire to be like the other person and to achieve success in the admiration condition. Thus, although closely related, elevation and admiration seem to elicit different physiological sensations and result in different motivational outcomes. However, this study was limited to adults and self-report measures and hence, did not assess participant’s prosocial behavior directly. This begs the question of how elevation and admiration develop in children and whether the specific emotional experience of elevation, in contrast to admiration, motivates individuals to engage in prosociality.

### Elevation as a motivator underlying prosociality

Evidence that elevation is a motivator of prosociality comes from prior work with adults suggesting that elevation leads to prosocial tendencies such as the motivation to become a better person (Haidt, 2000, 2003; Algoe and Haidt, 2009; Huta and Ryan, 2010; Aquino et al., 2011; Thomson and Siegel, 2013) and prosocial behaviors such as volunteering and helping (Haidt, 2000; Freeman et al., 2009; Schnall et al., 2010; Vianello et al., 2010; Aquino et al., 2011; Erickson and Abelson, 2012; Lai et al., 2014; Oliver et al., 2015; Siegel and Thomson, 2017). For example, Schnall et al. (2010) induced elevation in participants by showing them a 7-min video of people performing an act of moral goodness and kindness (the control group watched a nature documentary clip; the amusement group watched a comedy clip). Self-report measures revealed that participants in the elevation induced group felt more inspired, uplifted, and optimistic about humanity compared to the control group. Additionally, participants in the elevation group were more likely to help the experimenter by completing a boring task after the initial experiment than were the participants in the control group. Elevation inducing manipulations in studies with adult participants have also been shown to be associated with other positive outcomes including increases in charity donations (Freeman et al., 2009), volunteering (Cox, 2010) and decreases in racial biases (Lai et al., 2014). However, most prior work has compared elevation to either a control or an amusement condition (e.g., Schnall et al., 2010) and no study has compared elevation to the related other-praising emotion of admiration. Thus, it remains unclear whether the different motivational effects between elevation and admiration found by Algoe and Haidt (2009) result in actual differences in prosocial behavior.

Taken together, existing research on elevation has been solely conducted with adults and it remains unknown whether elevation, in contrast to admiration, leads to distinct motivational outcomes such as prosocial action. Thus, extending the adult literature by employing similar paradigms in school-aged children offers promising insights into the distinct role elevation might play in motivating prosociality at an age at which more complex social emotions and prosocial behaviors emerge.

### Evidence of elevation and admiration in childhood

To date, the developmental literature examining links between prosocial behavior and moral emotions has primarily focused on understanding the moral emotions that are elicited by a child’s performance of such behavior (Tracy and Robins, 2007; Aknin et al., 2012b; Vaish et al., 2016; Hepach et al., 2017b; Aknin et al., 2018, 2019; Van de Vondervoort et al., 2018; Vaish and Hepach, 2020; Gerdemann et al., 2022a; Vaish and Grossmann, 2022). For example, 2-year-old children show positive emotions after sharing resources with others (Aknin et al., 2012b), but also after seeing others receive the help they need (Hepach et al., 2012, 2013; Warneken, 2013; Grossmann et al., 2018; Hepach and Tomasello, 2020). However, one question that this prior work leaves open is whether simply witnessing a prosocial act, without being directly involved, elicits an emotion that promotes future helping behavior in children.

Initial attempts to explore the emotions elicited when seeing others being helped in children were conducted by Hepach et al. (2012) who measured 2-year-old children’s physiological arousal via their pupil dilation (i.e., as an indicator of distress) when they actively helped, when they observed a third-party helping, and when there was no help given to a person. Interestingly, the children showed similar pupil dilations when they could actively help themselves and when they observed a third party help compared to when no help was given. The consistent findings across the active and observational conditions led the authors to suggest that young children were satisfied seeing others being helped. Such insights suggest that young children are genuinely concerned about other’s wellbeing even if they cannot themselves provide the help and gain possible external rewards (e.g., social approval).

More recently Hepach and Tomasello (2020) measured the body posture of 4-year-old child dyads in a reward-collection task after which one of the two children needed more help across two conditions. The adult experimenter either helped the child who was in more need of help (deserving) or the child who was in less need of help (less deserving). Children’s upper-body posture after each outcome (i.e., deserving versus less deserving) was measured as an indicator of positively valenced emotions. The findings revealed that both children in the dyad displayed increased upper-body posture – including the child who did not receive a reward – when the more deserving child received help. In contrast, both children – including the child who received a reward – expressed decreased upper-body posture when the less deserving child received help. Thus, in both scenarios, both children displayed increased or decreased upper-body posture equally no matter whether they themselves were the less deserving child or not. Taken together, these findings suggested that positive emotions elicited in response to prosocial action are not restricted to one’s own actions and the possible gains [also see Gerdemann et al. (2022a)]. Children do not help to simply reduce their own personal distress or gain praise but likely do so because they are concerned for others’ wellbeing (Malti and Dys, 2015; Hepach, 2017;Hepach et al., 2017a,b; Malti, 2021).

While the above findings reveal that children show positive emotions when seeing others being helped, the studies were not designed to infer a specific emotion such as elevation. As such, the extent to which children experience specific emotions, such as feeling elevation when seeing others get the help they need and deserve and the extent to which children’s experienced emotions motivate future prosocial behavior, remain unclear. Further, positive emotions expressed in previous paradigms in response to seeing a peer receive deserving help (Hepach and Tomasello, 2020; Gerdemann et al., 2022a) might have served a signaling function, that is, the expression of positive emotions was exaggerated when a peer was present. Thus, more research assessing older children who have advanced introspection skills and can verbally express their emotional experiences in response to seeing excellence in others is needed. Finally, it is possible to explain past findings with children seeing a positive event which need not be specific to prosociality. Hence, including a condition unrelated to prosociality (i.e., admiration) is important to understand the distinct emotional characteristics of seeing excellence in others.

Besides body posture work with preschoolers, evidence around the development of both elevation and admiration is scarce. It is plausible that both emotions begin to develop when children start to understand social norms, rules, and standards of achievement, however, there is little research on elevation and admiration explicitly to support this point (Malti et al., 2020). However, recent evidence around *other-praising* moral emotions might shed some light onto the developmental onset of elevation and admiration. For example, respect (i.e., a positive feeling of admiration for other’s good behaviors) is a related emotion that recognizes the exceptional qualities of another (Schindler et al., 2013; Malti et al., 2020). Even though related, respect differs from admiration and elevation in that respect does not require the observed to be adored or liked and admiration and elevation do not necessarily imply to feel respect for another. Developmental evidence shows that respect is evident in middle childhood which aligns with the theory that other-praising emotions develop when children begin to have an understanding of fairness-based conceptions and an increasing awareness of other’s thoughts and feelings (Hsueh et al., 2005; Audley et al., 2020; Malti et al., 2020). Furthermore, awe is a related emotion that is elicited when seeing exceptional qualities not in people’s behavior but in expansive nature or art-related stimuli. Recent evidence suggests that awe is present in 4-to 9- (O’bi and Yang, 2024) and 8- to 13-year-old children (Stamkou et al., 2023) supporting the notion that middle childhood is a time when other-praising emotions emerge. Moreover, gratitude is an emotion elicited when actively receiving a good deed and is a gesture of appreciation and involves admiring someone else’s giving behavior. Prior research suggests that by 5 years of age, children display gratitude (Vaish and Savell, 2022) which becomes more sophisticated across the school years (Noles and McDermott, 2023). Finally, more negatively valenced emotions such as regret and shame, which are elicited in social contexts and require a reflection of one’s own behavior in accordance with global standards, are evident from 6 years onward also suggesting ‘that more complex social emotions develop in middle childhood (O’Connor et al., 2012; Gerdemann et al., 2022a; Gerdemann et al., 2022b). Thus, we conclude that middle childhood is a time when elevation and admiration likely emerge.

Taken together, both elevation and admiration involve the appraisal of another’s behavior based on either ethically-relevant (elevation) or non-ethically relevant (admiration) standards. Even though there is some developmental evidence suggesting that children show positive emotions in response to seeing someone help, the results from body-posture studies alone cannot infer a specific emotion such as elevation. More research with older children who can verbally express their emotional experiences and have advanced introspection is thus necessary. Further, based on recent evidence around other-praising emotions similar to admiration and elevation (e.g., awe, respect, gratitude), middle childhood seems to be the relevant time when more complex social emotions emerge due to children having a more sophisticated understanding of social norms, rules, and global standards which plays an important role in the development of other-praising emotions.

### Current study

The current study aims to address key gaps in the literature by exploring the specific emotional experiences and behavioral outcomes children display when seeing ethically-relevant (i.e., prosociality; elevation) and non-ethically relevant (i.e., talent; admiration) behavior in others. Specifically, the study is the first to assess both elevation and admiration in childhood and is thus advancing prior adult work around other-praising emotions as well as body posture work with preschoolers that cannot infer specific emotions. To explore the distinct role elevation might play in prosocial development, admiration offers a great contrast as research suggests that elevation and admiration are closely related (i.e., elicited when seeing excellence in others) but have distinct emotional and behavioral outcomes. So far, previous research investigating differences between elevation and admiration did not assess participants’ prosocial behavior but only their prosocial motivation (Algoe and Haidt, 2009). Thus, this study extends prior adult work by examining the distinct emotional and behavioral characteristics of elevation and admiration in middle childhood.

Moreover, prior work with adults has used self-report measures to assess the emotional characteristics of elevation and admiration and its links to adults’ behavior (e.g., Haidt, 2003; Algoe and Haidt, 2009), which contrasts with existing work with young children that has explored the emotional motivators of prosocial behavior using implicit (and arguably more objective) measures [e.g., eye-tracking to measure arousal as in Hepach et al. (2012); upper-body posture measurements as in Hepach et al. (2017b) and Hepach and Tomasello (2020)]. Although the use of implicit measures makes sense with young children since they likely have difficulty expressing complex emotional experiences compared to adults (Vaish and Hepach, 2020), they do not provide insights into their specific emotional experiences in such contexts. Hence, the current study was conducted with older children (i.e., 6.5- to 8.5-year-olds) so that we could implement a self-report measure of children’s emotional experiences and thus, bridge the gap between existing elevation work with preschool aged children (Hepach and Tomasello, 2020) and adults (e.g., Algoe and Haidt, 2009). Furthermore, developmental work around *other-praising* emotions (e.g., awe, respect) suggests that emotions of such complexity emerge during middle childhood, providing a justification for the middle childhood age range (Malti et al., 2020; Vaish and Savell, 2022; O’bi and Yang, 2024).

In a within-subjects design, 6.5–8.5-year-old children watched two video-clips (i.e., an elevation-inducing clip and an admiration-inducing clip) in a counterbalanced order. After each video, children rated their experiences of the five emotions that have previously been associated with elevation in the literature (i.e., happy, sad, warm and fuzzy, uplifted, caring) on a 7-point-scale. To shed further light onto the potential behavioral-feedback loop between elevation and prosocial behavior (Haidt, 2000; Fredrickson, 2001; Aknin et al., 2012a) and the distinct behavioral outcomes of elevation compared to admiration, we assessed children’s prosocial behavior after both videos by asking them if they would voluntarily answer algebra questions to donate food for people in need [i.e., Rice Game; see Meidenbauer et al. (2016)].

Our hypotheses were preregistered in OSF prior to data analysis (doi: 10.17605/OSF.IO/MK6EH; Project page: https://osf.io/vjkdp/) and were as follows: If elevation is a positive emotion with distinct emotional characteristics, then children should report higher scores for feelings associated with elevation (i.e., warmth, upliftment, care) after watching the elevation-inducing video, but not the admiration-inducing video. In line with prior work on children’s intrinsic motivation to see others helped and the emotion elicited (Aknin et al., 2012b; Hepach et al., 2012, 2017a, 2019), we expected the elevation condition to elicit a feeling of upliftment, warmth, and a desire to be caring (Haidt, 2000; Schnall et al., 2010) compared to the admiration condition. Secondly, in line with previous research on elevation and prosocial behavior (Freeman et al., 2009; Cox, 2010; Schnall et al., 2010; Thomson and Siegel, 2013, 2017; Lai et al., 2014), we expected that induced elevation, but not admiration, would lead to increased prosocial behavior in children. Specifically, we hypothesized that children would engage with the prosocial game longer after the elevation condition compared to the admiration condition.

## Methods

### Participants

Participants were 182 6.5-to 8.5-year-old children (see Table 1 for demographic information). Children were part of a larger longitudinal project on the ontogenetic roots of cooperative understanding and ability (ORCA) at the University of Auckland [see also Breeland et al. (2022) and Gibhardt et al. (2024)]. Data for this study came from the last data collection wave (DCW) when children were approximately 7 years old. The main sample has been participating in ORCA since they were 9 months old (*Original*; *n* = 131). We recruited an additional sample (Booster; *n* = 52) to boost our sample size as the original sample at DCW1 included 255 participants and we lost participants due to attrition (e.g., COVID-19). Some of the other tasks in the session required a larger sample size and thus, a booster sample was recruited during DCW7 to participate in a peer-cooperation task with a same-aged same-gender ORCA participant. For the data presented in this manuscript, a power analysis demonstrated that 57 participants were needed to detect a medium effect size (Cohen’s *d* = 0.5) in a Wilcoxon-Signed Rank test with matched pairs (*α* = 0.05, *β* = 0.95).

**Table 1:** Demographic information for the original and booster samples.

Demographic variables	Original		Booster	
	*M*(*SD*)	Frequencies	*M*(*SD*)	Frequencies
Sample size (*n*)		130		52
Age (months)	84.55 (6.33)		83.23 (3.93)	
Gender		Girls = 61		Girls = 26
School attend (months)	23.03 (7.95)		21.31 (6.10)	
Child ethnicity (%)				
Pasifika		77%		0%
Other European		77%		3.85%
Asian		77%		7.69%
NZ European and Other		77%		1.92%
Other European and Asian		77%		0%
NZ European and Pasifika		2.31%		0%
NZ European and Other European		5.38%		3.85%
NZ European and Māori		6.92%		1.92%
NZ European and Asian		9.23%		13.46%
NZ European/Pākeha		69.23%-		55.77
Missing (NA)		–		3.85%

Child ethnicity as reported by parent report. “Other” includes South African, British, Irish, and Indian. Missing (NA), parent did not provide demographic information.

We received approval from the University of Auckland Human Ethics Committee to invite these “peers” from DCW7 to participate in DCW8, which did not involve any peer related tasks. The DCW8 session lasted for 1.5- to 2-h. Criteria for exclusion from the final sample of the ORCA project included: cognitive or language impairments diagnosed during the course of the project. In the current study, we excluded one additional child from the final sample due to a diagnosis of developmental delay and epilepsy.

Families were recruited through public fairs, social media, or word of mouth. As a token of appreciation, children received a small toy at the end of the session and parents went into the draw to win grocery or shopping vouchers. All procedures were approved by the University of Auckland Human Participation Ethics Committee.

### Design

In a within-subjects design we exposed each child to an elevation condition and an admiration condition with the order counterbalanced across children. Since this project was part of a larger longitudinal study, the first condition took place at the end of the first part of the session prior to a break (i.e., Part 1 that occurred approximately 40 min into the session). The second condition (Part 2) took place at the end of the session approximately 45 min after Part 1. The order for each part was identical with one exception, before Part 1, children participated in the emotion training phase. The procedure for each part (i.e., Part 1 and Part 2) consisted of the following phases: baseline emotion phase, elevation/admiration inducing story phase, elevation/admiration video clip, emotion test phase, and voluntary prosocial game.

### Materials and procedure

At the beginning of the session, the main experimenter (E) met the caregiver and child at the carpark and walked them up to the research space. Once children arrived in the lab, E outlined the tasks and completed consent procedures. While the caregiver completed the consent form, E played with the child and completed assent procedures with the child. Once the caregiver completed the consent forms, the child gave assent and felt comfortable, the session began with E walking the child to the adjacent testing room. During the study, caregivers were encouraged to stay in the waiting room area where they could view the session via a closed-circuit TV. In instances in which the caregiver stayed in the room (e.g., due to the child’s request), E asked the caregiver not to interfere with the session. The child completed a series of tasks that were completed in the prior DCW of the longitudinal study. Only the tasks and materials directly relevant to the present study are described below.

#### Emotion training phase

**Figure 1:**
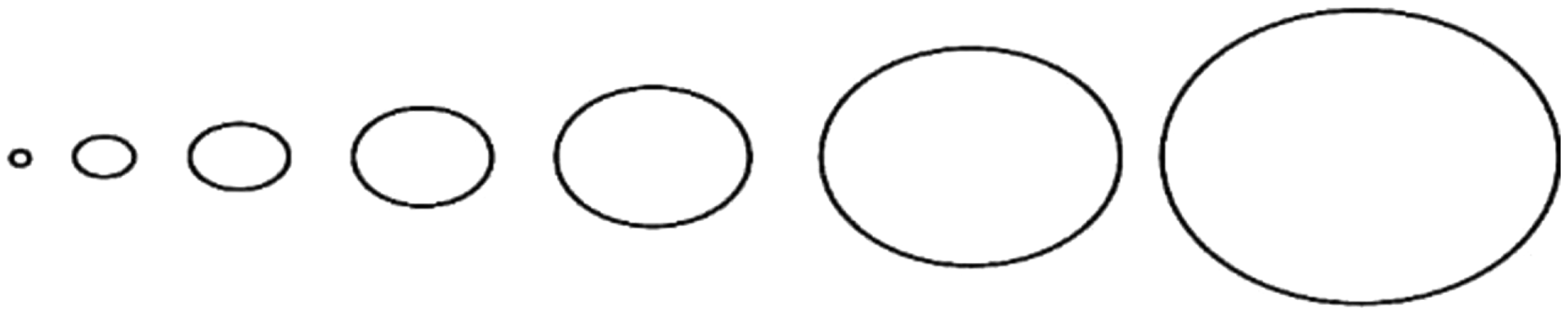
Circle scale. Circle scale for children’s emotion self-report. The small circle **(left)** was introduced as not feeling the emotion at all and the big circle **(right)** was introduced as feeling a huge amount of the feeling. The scores applied ranged from 1 (not at all) to 7 (a huge amount).

E introduced children to the 7-point-circle scale (see Figure 1) and trained children to use the scale to describe the intensity of three emotions represented three emoji faces (happy, sad, and neutral). After placing the circle scale on the table, E said “Look there are seven circles here. They go from this smallest circle which means not at all, to the middle one which means a little bit, to the biggest one which means a huge amount.” E then placed the one happy emoji, one neutral emoji, and one sad emoji face on the table and said “Look, I’ve got three faces here. This face feels happy, this face feels ok, and this face feels sad (the order of the emoji was counterbalanced across children). Dependent on the counterbalancing script, E either asked children how happy or how sad each face is feeling. Thus, when asked how happy the happy face is, children should select the biggest circle, when asked about the sad face, children should select the smallest circle, and when asked about the neutral face children should select a circle in the middle of the scale. If children selected correctly, E moved on to the next face. However, if children selected the incorrect circle (i.e., big circle for sad face), E reminded the children what the circles meant.”

#### Baseline emotion phase

Following the training phase, E presented children with the circle scale and an additional 5 emoji faces on a Samsung tablet (see Supplementary Material S1) and said, “Look I have got some more faces about how people can feel on this tablet.” Consistent with previous work (Haidt, 2000, 2003), children were presented with four emoji faces that displayed elevation related emotion expressions (i.e., happy, uplifted, warm and fuzzy, and caring). A fifth emoji depicting a sad face was also shown to children as a negatively valenced control emotion. During this phase, E showed children each emoji and explained the emotional experience that is typically associated with that emoji expression (see Figure 4 for the explanations for emoji). Immediately following each explanation, E asked the child how much of the emotion the child was feeling right now on the circle scale, which served as the emotion baseline measure.

#### Elevation/admiration inducting story phase

To assist children with their understanding of the elevation/admiration inducing video events, E presented the children with a summary of the story using screenshots of key moments in the videos on a Samsung tablet (see Supplementary Materials S2, S3). The materials for the elevation inducing story and video involved six screenshots of a YouTube video labeled ‘Pub Fosterhjem: A child has nothing to eat at school’.^1^ Previous work around elevation in adults presented participants with video clips that involved adults (e.g., Oprah donating money; e.g., Schnall et al., 2010). To elicit elevation in children, we chose a video involving children sharing and a classroom context because such a setting should be more familiar and relatable to 6.5- to 8.5- year-old children.

The materials for the admiration inducing story and video involved four screenshots of a YouTube video from the “Kids are awesome” series.^2^ We chose this video because it showed children at a similar age (i.e., middle childhood) performing talented acts. Both video clips were 1 min long and were edited to have similar neutral music.

Following the story, E asked the child three comprehension questions to ensure that the child understood the storyline. In the elevation condition, E asked the child the following three questions: “At the beginning of the story, was the boys lunchbox empty or full?” (Correct answer: empty). “At the end of the story, what was in the boys lunchbox?” (Correct answer: food). “How did the food get into the lunchbox?” (Correct answer: the other children shared). In the admiration condition E asked: “What was this story about?” (Correct answer: talented children). “What did the children do in the story?” (Possible answers: handstands, golf, gymnastics). “Can you remember what happened at the end of the story?” (Correct answer: a boy was riding a unicycle). If children did not provide the correct answer or could not remember, E told them the answer and asked the question again as a final comprehension check. All children in this study got at least two comprehension questions correct.

#### Emotion test phase

Immediately after the video, E presented the child with the circle scale and asked if the child remembered what the circles meant. If the child said no, E re-stated the meaning of the circles as per the training phase. E then presented the child with each of the five emoji faces on the Samsung tablet again and asked the child to indicate on the circle scale how much of the given emotion they experienced at the end of the video. For example, for the warm and fuzzy emoji, E asked “At the end of the video, how warm and fuzzy did you feel?” E specifically asked how the child was feeling at the end of the video to increase the likelihood that children would consider the critical event in the elevation video where other children shared with the boy when making their decision.

#### Voluntary prosocial behavior phase (Rice Game)

After the emotion test, E told the child that it was almost break time (Part 1)/finished (Part 2). E further stated: “I only have one more game that you can play before you take your break (Part 1)/you are finished for today (Part 2)” and showed the child a screenshot of the Rice Game on the Samsung tablet. The Rice Game is a quiz available on the internet. We chose the basic algebra option in which children had to answer basic math questions that progressively got harder depending on the child’s performance. Children played the game on the Samsung tablet. To introduce the game E said, “This game is called the Rice Game and the Rice Game is a type of Quiz game where people answer questions. But what’s special about this game is that for every right answer, food will be given to children all over the world who do not have enough to eat. So, the more questions you answer, the more food will be given to children who need help.” E then asked the child to indicate on the circles how much they would like to play the game. If the child chose the smallest circle (not at all), they immediately took their break/finished. If the child chose any of the other circles, E opened the Rice Game App on the tablet with the first math question displayed on the screen, turned the screen to the child, and said: “This is the game. Look, this is the question (e.g., read out 7 + 5) and here are the possible answers (E reads out, e.g., 10, 11, 12, 16). When you find the right answer, you choose it by tapping on it like this.” E then tapped the right answer and said: “And look, food is put into the bowl down here.” E pointed to the bowl with food and further states: “In this game, you can fill up as many bowls as you like. And at the end of the game, actual food is sent to a child who needs it.” Following this, E hid the tablet under the table to ensure that the child will pay attention to the following statement: “You can decide how many questions you want to answer before you take a break. You do not have to answer any, but if you want to, you can answer as many questions as you want. And remember, the more questions you answer, the more food is put into the bowls. You can fill up as many bowls as you like. I’ll be out there doing some work (E points to the door) and when you are done you can let me know by calling “finished” and we can take a break/finish okay?.” E passed the tablet to the child, started the stopwatch, and left the room. There were no cases in which a child was not comfortable with being in the room alone.

The maximum duration for the Rice Game was 10-min. If children hit the 10-min mark, E would enter the room, stop the task, and say, “Let us take a break!” or “You are all done for today!.” In instances where the child asked E for help during the game, E replied that they were doing work and that the child could play for as long as they wished, thereby not providing any help except in cases where children asked what a given symbol meant (e.g., *x* means multiply).

### Scoring, coding and variable construction

#### Emotion self-report

Children received two scores for each emotion presented during the baseline and test phases for each condition (i.e., elevation, admiration). Scores ranged from 1 to 5 corresponding to the circles on the circle-scale (1 = smallest circle, 7 = largest circle). Thus, lower scores reflected no to some experience of the emotion and higher scores reflected a lot to a huge experience of the emotion (see Table 2 for descriptive statistics).

**Table 2:** Descriptive statistics for children’s baseline and test emotion report and Wilcoxon signed rank test results for children’s difference scores across conditions.

Outcome variable	Elevation			Admiration			Wilcoxon signed rank test		
	*M(SD)*			*M(SD)*					
	BL	*T*	Diff. score	BL	*T*	Diff. score	*Z*	*p*-value	*N*
Happy	6.16 (1.30)	6.12 (1.57)	**−0.07 (1.68)**	6.20 (1.15)	5.85 (1.63)	**−0.35 (1.66)**	**2.13**	0.033	181
Sad	1.36(0.98)	1.67 (1.40)	0.31 (1.39)	1.42 (1.04)	1.52 (1.40)	0.10 (1.42)	1.58	0.115	182
Uplifted	5.18 (2.07)	4.16 (2.32)	−1.02 (2.37)	4.95 (2.14)	4.95 (2.17)	−0.02 (1.95)	**−4.94**	<0.001	181
Warm	4.82 (2.14)	4.93 (2.17)	**0.11 (2.02)**	4.68 (2.19)	3.92 (2.36)	**−0.78 (2.19)**	**4.06**	<0.001	181
Caring	5.30 (1.94)	5.85 (1.71)	**0.55 (1.70)**	5.27 (1.99)	4.66 (2.24)	**−0.61 (2.08)**	**5.78**	<0.001	180

Table depicts mean (M) and standard deviations (SD) for children’s emotion baseline (BL), test (T), and difference scores (Diff. Score) each emotion across conditions. Wilcoxon-Signed Rank Test results show the differences in difference scores between conditions. Difference scores were calculated as follows: Emotion Test Score – Emotion Baseline Score. Significant values are marked in bold.

#### Rice Game performance

Children’s performance during the Rice Game was coded offline by the lead author. The total duration (minutes) children played the Rice Game on a frame-by-frame basis was coded using the software, Interact (Mangold Interact Software). For each frame, the coder indicated if the child was engaged or disengaged. Engagement included the child actively playing the game, for example, by calculating math problems in their head or tapping on answers on the tablet. Disengagement was indicated when the child did not appear to be actively playing the game and thus involved behaviors such as looking or walking around the room, trying to get attention from the experimenter by talking about a subject unrelated to the Rice Game, or randomly tapping on the tablet. A second reliability coder coded 30% of the total sample (55 total: 28 Original and 27 Booster). Cohen’s Kappa and ICC values for engagement were high: κ = 0.925; ICC = 0.986 [0.980, 0.990]. For the main analyses (i.e., survival modeling), the amount of time (minutes) that children were engaged in the Rice Game before either becoming disengaged for more than 5 s, or reaching the 10-min cut-off mark, was calculated. That is, children who played the Rice Game for 10 min but were disengaged for more than 5 s at the 4-min mark, received an engagement score of 4 min. This allowed us to capture how long children stayed engaged in the Rice Game before losing interest.

The number of correct and incorrect responses children provided was coded offline in a separate coding wave to provide a measure of accuracy. A second coder blind to the hypotheses coded 30% of the overall sample (55 total: 28 Original and 27 Booster) for reliability. Coding involved correct and incorrect responses for each condition. Cohen’s Kappa’s for accuracy codes were high: κ = 0.959 (correct responses) and κ = 0.956 (incorrect responses).

### Analysis plan

The goals of the present research were to (1) assess the specific emotional characteristics elicited in children when seeing prosociality in others (elevation) versus seeing talent in others (admiration) and (2) test whether seeing prosociality in others leads to increased prosocial behavior in children compared to seeing talent. To fulfill these goals, we first conducted preliminary analyses to test differences in our outcome measures for the following variables: sample (Original and Booster), face cover (mask vs. no mask), age, months attended school, order of condition, and gender. Visual inspection (i.e., histograms) revealed that our outcome variables did not follow normal distributions and hence, we used non-parametric tests. Thus, we explored differences between baseline and test emotion self-reports conducting Wilcoxon-Signed Rank analyses using SPSS (Version 27). Further, for our main analyses assessing the differences specific emotional characteristics (e.g., whether elevation or admiration increased/decreased for a specific emotion), we calculated difference scores by subtracting the baseline scores from the test scores for each emotion across conditions. We corrected for multiple testing applying the Bonferroni method.

For the Rice Game measures, we chose a survival modeling approach in R (version 4. 2. 2) to test whether elevation, in contrast to admiration, leads to increased prosocial motivation. We used the packages *Survival* and *Coxme* (Therneau, 2015; Therneau and Lumley, 2015), to calculate the likelihood children stayed engaged in the task over time between conditions. Finally, to test whether children’s accuracy (correct vs. incorrect responses) differed between conditions, we calculated a Generalized Mixed Model with binomial error structure and random intercept (repeated measures) in R using the function glmer (package lme4; Bates et al., 2015).

## Results

### Preliminary analyses

Preliminary analyses revealed no differences in our outcome variables between the original and booster samples. Furthermore, because DCW8 was completed during COVID-19 restrictions in New Zealand, there were inconsistencies across sessions in whether the experimenter was required to wear a mask. Mann–Whitney *U* tests revealed no differences for the emotion baseline and test phases between children who had an experimenter who wore a mask (*n* = 76) and did not wear a mask (*n* = 106) in the admiration baseline and test conditions (see Supplementary Material for a more detailed overview).

Moreover, we tested whether there were differences in our outcome variables dependent on the order (i.e., counterbalancing, Part 1 vs. Part 2) children were presented with the conditions. First, Mann–Whitney *U* tests showed no differences for order in the admiration condition for children’s emotion self-reports. Second, preliminary analyses revealed a difference depending on order in the elevation condition for children’s baseline happiness with children reporting higher BL happiness scores in Part 2 compared to Part 1. There were no differences in order for the remaining baseline and test emotion reports in the elevation condition. Finally, there were no differences for order in the Rice Game measures for the neither the admiration nor the elevation condition (see Supplementary Material for a more detailed overview).

Since our sample included a relatively wide age gap and the Rice Game involved math questions, we ran spearman correlations to see whether the child’s age or months spent attending school were associated with our Rice Game measures. There were no significant associations between the child’s age and Rice Game engagement in the *Admiration* [*r*s(179) = −0.035, *p* = 0.636] or *Elevation* [*r*s(176) = 0.074, *p* = 0.323] conditions. There was no significant association between the child’s age and accuracy in the *admiration* condition [*r*s(170) = 0.130, *p* = 0.089]. However, there was a positive correlation between age and Rice Game accuracy in the *elevation* condition suggesting that in the elevation condition, older children were more likely to have a higher number of correct responses [*r*s(168) = 0.223, *p* = 0.003]. We thus controlled for age in our main analyses. Furthermore, there was no association between school months and Rice Game engagement in the *admiration* [*r*s(176) = −0.009, *p* = 0.910] or *elevation* [*r*s(173) = 0.008, *p* = 0.917] conditions nor between school months and Rice Game accuracy in the *admiration* [*r*s(167) = −0.022, *p* = 0.776] or *elevation* [*r*s(165) = 0.016, *p* = 0.833] condition.

In terms of gender differences, Mann–Whitney *U* tests revealed significant gender differences in the *elevation* condition for the emotion baseline measure. Specifically, during the emotion baseline phase before the elevation video, girls reporting higher feelings of care and lower feelings of upliftment compared to boys, *Z* = −2.64, *p* = 0.008 and *Z* = 2.40, *p* = 0.016, respectively. Critically however, there were no gender effects for the Rice Game and emotion test self-report measures.

Finally, Wilcoxon-Signed Rank tests revealed no differences in baseline emotion reports for each emotion across conditions (see Supplementary Material for a detailed overview).

#### Does seeing prosociality elicit specific emotional characteristics in young children?

**Figure 2:**
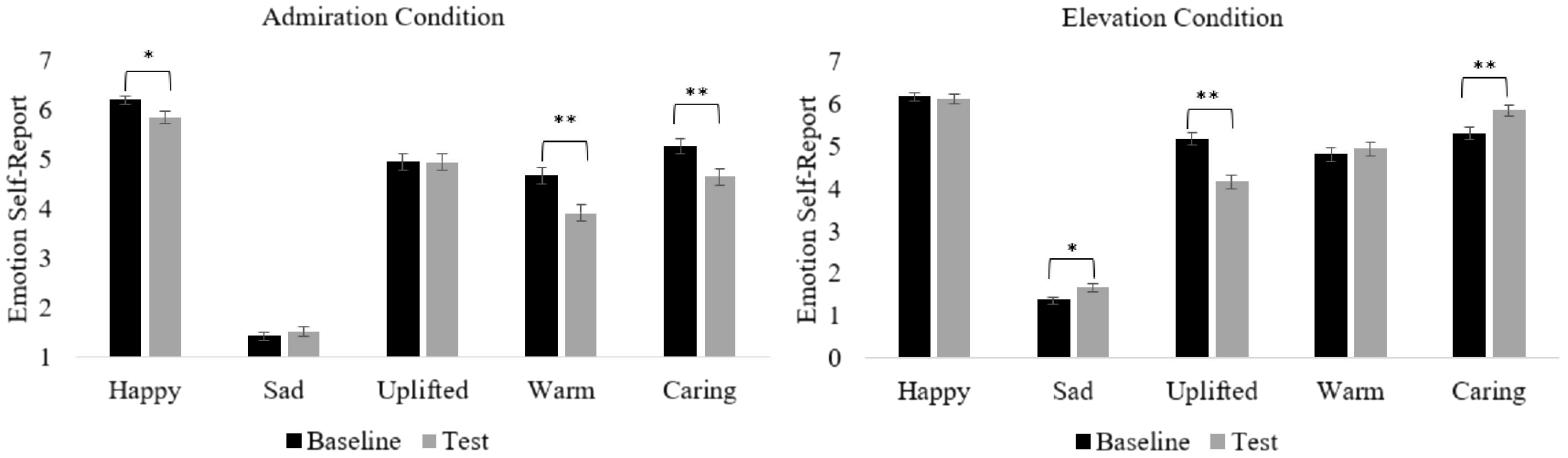
Contrasts between baseline and test emotion self-report means (±SE) for each condition. Figure shows baseline and test emotion self-report mean (±SE) differences across conditions. The Bonferroni correction was used to control for multiple testing (0.05/2=0.025). SE, standard error. ^*^P < 0.025, ^**^p < 0.01.

For each of the five emotions, we tested whether (1) children’s baseline and test emotion self-reports differed within condition (i.e., *Baseline vs. Test*; see Figure 2) and (2) whether there were differences in children’s scores between conditions. Main analyses to test differences between conditions were calculated using children’s difference scores. Table 2 provides a summary of the descriptive statistics and the Wilcoxon-Signed Rank test results for difference scores between conditions.

##### Happiness

###### Baseline vs. test

There was a difference between baseline and test emotion self- reports in the admiration, but not the elevation, condition. Children reported slightly lower happiness in the admiration test phase compared to baseline.

###### Differences between conditions

Wilcoxon-Signed Rank tests revealed a significant difference in differences scores for happiness between conditions. Specifically, children reported lower happiness in the admiration condition compared to the elevation condition.

##### Sadness

###### Baseline vs. test

Findings revealed an increase in sadness in the test phase compared to the baseline phase in the elevation condition. There was no significant difference between baseline and test in the admiration condition.

###### Differences between conditions

Analyses revealed no significant difference in sadness emotion reports between conditions.

##### Upliftment

###### Baseline vs. test

Children’s report of feeling uplifted decreased significantly in the elevation test emotion phase (relative to baseline) but there was no difference in the admiration condition.

###### Differences between conditions

Wilcoxon-Signed rank tests revealed a significant difference in children’s self-report of feeling uplifted between conditions. Specifically, children reported significantly higher levels of upliftment in the admiration condition compared to the elevation condition.

##### Warm and fuzzy

###### Baseline vs. test

Children’s levels of feeling warm and fuzzy significantly decreased in the admiration test phase compared to baseline. There was no difference in children’s self-reports of warmth in the elevation condition.

###### Differences between conditions

Results revealed a significant difference in warmth between the elevation and admiration conditions. Difference scores revealed higher levels of warmth in the elevation condition compared to the admiration condition.

##### Care

###### Baseline vs. test

There was a significant difference in difference scores between conditions, that is, children reported lower levels of care in the admiration test emotion phase (relative to baseline) but reported higher levels of care in the elevation test emotion phase (relative to baseline).

###### Test emotion self-reports

Children reported significantly higher levels of care in the elevation emotion test phase compared to the admiration emotion test phase.

In sum, findings revealed that elevation and admiration elicit different *emotional phenotypes*. Specifically, children experienced higher levels of happiness, warmth, and care in the elevation condition compared to the admiration condition. In addition, children reported higher levels of upliftment in the admiration condition. Since the strongest effects were found for upliftment, warmth, and care, we conducted follow-up partial correlations to check whether the pattern of a specific emotion could be explained by the other emotions. Interestingly, for the elevation condition, findings revealed significant partial correlations between upliftment and warmth when controlling for care (*r* = 0.181, *p* = 0.015) and between care and warmth controlling when controlling for upliftment (*r* = 0.175, *p* = 0.019), but not between upliftment and care when controlling for warmth (*r* = −0.057, *p* = 0.446). Conversely, we found a significant partial correlation between care and warmth when controlling for upliftment in the admiration condition (*r* = 0.313, *p* < 0.001), but not for upliftment and warmth controlling for care (*r* = 0.047, *p* = 0.533) or upliftment and care when controlling for warmth (*r* = 0.143, *p* = 0.056). These findings further suggest that a different emotional response was triggered across the elevation and admiration conditions.

#### Does elevation motivate prosocial behavior in children?

##### Rice Game engagement

**Figure 3:**
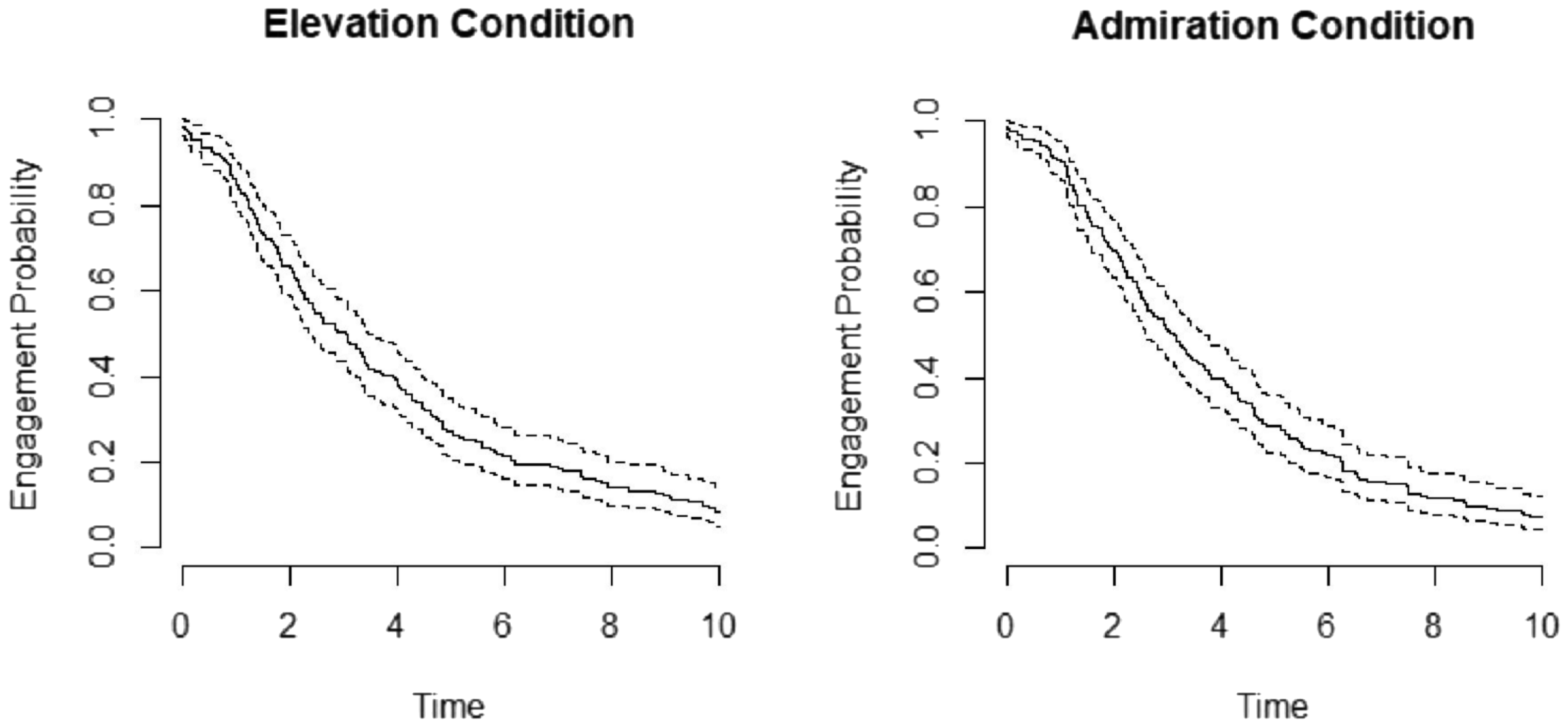
Survival modeling curves for each condition. Figure shows the probability of children staying engaged in the rice game over time for the elevation and admiration condition.

To test whether condition influenced the time that children engaged in the Rice Game, we implemented a survival modeling approach in R. A survival model allowed us to plot the differences in the probability of children staying engaged in the Rice Game over time between conditions (see Figure 3). Using the R package *coxme*, we compared a full model including our main predictors (i.e., condition, age, gender), as well as a random intercept for subject to a null model excluding condition. There was no statistically significant effect of condition on children’s likelihood of being engaged in the Rice Game, *β* = 0.07 +/− 0.12, *χ^2^*(1) = 0.37, *p* = 0.542. In addition, there were no effects of age, *z* = −1.42, *p* = 0.16, or gender, *z* = −0.44, *p* = 0.66.

**Figure 4:**
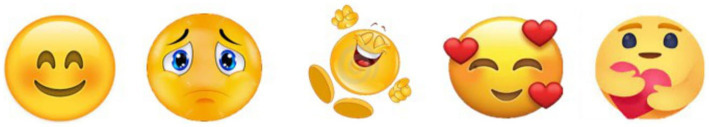
Emotions from left to right: happy, sad, uplifted (“This face is feeling uplifted, for example, a child might feel uplifted when they have a fun playdate with a friend.”), warm and fuzzy (“This face is feeling warm and fuzzy, for example, a child might feel warm and fuzzy when they get a big hug from their mum or dad.”), caring (“This face is feeling caring. A child might feel caring when they see a friend crying because they hurt themselves.”). Source: https://mondaymandala.com/printable-emojis/.

##### Rice Game accuracy

Next, we tested the effect of condition on children’s proportion of correct responses using a Generalized Linear Mixed Model (GLMM) approach in R. The number of correct and incorrect responses was a count response and thus we calculated a GLMM with binomial error structure including a random intercept for subject. The model included condition as a fixed effect as well as a random slope and children’s age and gender were included as control variables. Results revealed no effect of condition on Rice Game accuracy, *b* = 0.04, *se* = 0.07, *z* = 0.55, *p* = 0.582. In addition, there was no effect of gender, *b* = −0.05, *se* = 0.12, *z* = −0.42, *p* = 0.672. However, there was a significant effect of age (*b* = 0.14, *se* = 0.056, *z* = 0.254, *p* = 0.011), with older children having higher accuracy scores.

In sum, analyses provided no evidence that seeing prosociality in others leads to children’s increased Rice Game engagement and accuracy (i.e., prosocial motivation) compared to seeing talent in others (Figure 3).

## Discussion

The aim of the current study was to investigate the specific emotional characteristics and behavioral outcomes of seeing prosociality versus talent in 6.5- to 8-year-old children. By doing so, this research addresses a key limitation in previous work around the moral emotion of elevation, which is that it has been solely conducted with adults. Comparing elevation to another *other-praising* emotion (i.e., admiration) allowed us to extend prior findings with adults which suggest that elevation differs in emotional and motivational effects compared to admiration (e.g., Algoe and Haidt, 2009), and leads to increased prosocial behavior compared to a neutral condition and an amusement condition (e.g., Schnall et al., 2010). Furthermore, most prior developmental work suggests that young children experience positive emotions after helping themselves (Aknin et al., 2012b; Hepach et al., 2017b) and after seeing someone else help (Hepach et al., 2012; Vaish and Hepach, 2020) but do not shed light onto the nature of these emotions. Thus, the current study extends previous developmental work around the emotional motivators underlying prosociality by showing that elevation and admiration elicit distinct *emotional phenotypes* but no differences in prosocial motivation. Overall, the findings in the current study revealed mixed support for our hypotheses and are discussed below with respect to the key research questions.

### Evidence for distinct emotion-characteristics of elevation in children

As expected, and consisted with prior literature, the elevation condition elicited higher feelings of happiness, warmth, care, and sadness in children compared to the admiration condition (Haidt, 2000, 2003; Algoe and Haidt, 2009). While these findings are consistent with prior work, they extend previous work with adults by showing that young children experience similar emotions when seeing prosociality in others. Furthermore, identifying the specific emotional experiences elicited when observing ethically-relevant excellence versus non-ethically relevant excellence extend prior developmental work showing that children show positive emotions when seeing others being helped by shedding more light onto the specific positive emotional experiences that preschoolers might experience (Hepach and Tomasello, 2020).

Regarding children’s self-report of experiencing happiness, our findings reveal that observing talent led to children reporting lower levels of happiness than observing a child being helped. Lower levels of happiness after watching the admiration video compared to the elevation video may have been due to children experiencing feelings of inferiority, insecurity, or jealousy (van de Ven et al., 2011). Precisely, seeing other talented children might elicit insecurities as children might feel reminded that they cannot do the same things as children in the videos which is consistent with research showing that admiration and envy are closely related (van de Ven, 2017). Indeed, during the session, the experimenter noted that some children commented that they could not do *any* of the things the other children did. Thus, in contrast to elevation which we expected, and other evidence suggests, is likely to elicit other-oriented concern and a pleasant feeling, admiration may be more likely to elicit self-oriented thoughts such as social comparisons (van de Ven, 2017).

Further support for the possibility that admiration may induce self-oriented thoughts and elevation may induce other-oriented thoughts comes from our finding that feelings of care increased in the elevation condition but decreased in the admiration condition. The elevation video likely led children to want to care as well (other-oriented concern) whereas admiration would not have involved such other-oriented thoughts, but instead likely induced self-oriented thoughts a such as “I want to excel at something, too” (Silvers and Haidt, 2008; Algoe and Haidt, 2009; Schnall and Roper, 2012).

Moreover, findings revealed that children reported significantly higher levels of upliftment in the admiration condition compared to the elevation condition. This is in contrast to prior work (Haidt, 2003) suggesting that elevation leads to increased upliftment in adults. There are several possible explanations for this unexpected finding. First, children might associate the word *uplifting* with excitement or high energy as some children in the baseline emotion phase referred to feeling uplifted when they received a gift from a friend. Prior work suggests that admiration is associated with an increased heart rate and high energy (Algoe and Haidt, 2009) and thus, it is possible that the difference in upliftment between conditions is due to children’s interpretation of the word *upliftment*. In contrast, adult participants in previous studies likely associated upliftment with feeling inspired (Haidt, 2000, 2003) which is more likely elicited when seeing an elevation inducing stimulus compared to an admiration inducing stimulus. Therefore, whereas feelings of upliftment related to elevation might be more inspirational and low energy, admiration likely leads to feelings of upliftment closely related to excitement (e.g., increased heart rate). Thus, the children in this study might have linked upliftment to excitement rather than inspiration.

Second, it is possible that the difference in upliftment between conditions is due to our stimulus video. The elevation video was selected as the stimulus for this initial investigation of elevation in children because it shows children helping a person in need (i.e., moral excellence) which we thought would be likely to induce a sense of upliftment in children. However, it is possible that seeing a child in need in this context is not uplifting for children. Indeed, our findings suggest that elevation elicits higher sadness in children which is likely due to seeing a child in need in the beginning of the video. This might explain why children report lower levels of upliftment in social contexts that elicit empathy (i.e., feeling a distressed feeling when seeing someone in distress) such as in the elevation condition. Interestingly, the relation between positive emotions such as warmth and care and sadness may have a temporal relationship such that sadness could help induce warmth and care via an empathetic process. However, whereas warmth and care are calm sensations, upliftment is more associated with high energy and excitement which is a stark contrast to sadness. Indeed, research suggests that seeing another in need elicits arousal (i.e., negative emotions) which decreases when they see that person being helped (Hepach et al., 2012). In the current study, children may have felt an increase in arousal at the beginning of the elevation video when seeing the boy in need, but this arousal may have decreased after his class helped him. Thus, at the end of the clip children might have felt a sense of relief or a calming sensation (e.g., warmth, care) more so than a feeling of upliftment (i.e., high energy, excitement).

Although our findings supported our hypothesis that warmth would be higher in the elevation condition compared to the admiration condition, examination of the difference scores between children’s baseline and test emotion self-reports revealed that the difference in warmth between conditions was likely due to a *decrease* in the admiration condition, not an *increase* in the elevation condition. Our findings that seeing talent in others does not elicit warmth in the chest is consistent with prior research with adults (Algoe and Haidt, 2009) and this is the first evidence of decreases in warmth after watching other talented children. One explanation might be that admiration is associated with increased heart rate and high energy whereas a feeling of warmth might be associated with low energy.

In contrast to previous work (e.g., Haidt, 2000), our findings showed a slight increase in warmth in the elevation test phase compared to the elevation baseline phase but this effect was not significant. One explanation might be that children were confused during the baseline phase regarding where to base their rating on. Specifically, in the current study, even though the experimenter asked children how much of an emotion they are feeling in this moment, the experimenter provided an example for each emoji face, for example, “You might feel warm and fuzzy when you get a hug from your mum and dad.” It might be that children based their responses on a past event instead of how they are feeling in this moment. Thus, the baseline measurement, even though providing an indication of the direction of the emotion experience, is to be considered with some caution because children might have limited introspection about emotion experiences in the present moment. Emotion baseline ratings were generally quite high and thus, it remains unknown whether self-report measures with young children adequately capture children’s emotion experiences. Future investigations into the specific emotional experiences of seeing others being helped versus talent might further explore children’s reasoning behind each emotion, for example, by asking children why they felt a certain emotion in a given context. This could clarify whether children based their baseline emotion ratings on the moment or on an imagined scenario in the past where they felt the emotion intensely.

Taken together, findings of the current study extend prior work with adults and suggest that elevation, in contrast to admiration, is generally associated with higher feelings of happiness, warmth, care, and sadness in 6.5-to 8.5-year-old children (Haidt, 2000, 2003). Admiration, on the other hand, is associated with feelings of upliftment in 6.5-to 8.5-year-old children, which most likely links to high energy and increased heart rate evident in adult research (Algoe and Haidt, 2009). Since this was the first study to design a paradigm to assess elevation in children using a self-report measure to investigate the nature of specific emotions, future research in this area might use additional physiological measures such as eye-tracking (i.e., arousal), heart rate, and body posture [i.e., Kinect technology; see Hepach et al. (2012, 2017b)] to supplement and gain a more sophisticated understanding of children’s emotions.

### Elevation did not lead to increased prosocial behavior

Surprisingly, our findings did not provide evidence of increased engagement or accuracy in the Rice Game in the elevation condition compared to the admiration condition suggesting that elevation does not lead to increased prosocial behavior in 6.5-to 8.5-year-old children. This is the first study to test the extent to which inducing elevation, compared to inducing admiration, leads to increased prosocial behavior in children, but these findings are inconsistent with adult literature which suggests that inducing elevation (in contrast to a neutral or amusement condition) motivates prosocial behavior in adults [e.g., Landis et al., 2009; Cox, 2010; Schnall et al., 2010; Lai et al., 2014; for a review see Thomson and Siegel (2017)].

One reason for the lack of significant differences between the two conditions in the present study might be due to the comparison condition that we opted to include in this study. In contrast to prior work who implemented control and amusement conditions to test whether the elevation condition led to increased prosocial behavior (Schnall et al., 2010), we selected an admiration condition because elevation and admiration have been theorized to be closely related (i.e., other-praising emotions) but different in their motivational effects. Specifically, prior work with adults suggests that elevation, in contrast to admiration, leads to increased prosocial motivation (Algoe and Haidt, 2009). However, prior work has only provided evidence that elevation, in contrast to admiration, has distinct motivational, but not, behavioral effects. Perhaps surprisingly, our findings suggest that elevation and admiration have similar behavioral outcomes. One explanation for this is that both elevation and admiration may motivate children to excel and be a *better* person and help. Since admiration is also part of the other-praising emotions it might elicit a general desire to be a *good* person which involves engaging in prosocial behaviors. Alternatively, seeing children who are talented might elicit a desire to also excel at a task and the Rice Game offered an opportunity for children to excel at math (Algoe and Haidt, 2009; Immordino-Yang et al., 2009). Thus, the Rice Game offered the opportunity to both, be prosocial and to show one’s ability at a task. We encourage future research to include a control condition to test whether children’s engagement and accuracy scores are due to both conditions eliciting prosociality or whether it is due to children’s general enjoyment of the task.

Another possible explanation for why admiration and elevation elicit similar prosocial motivations is that admiration is like awe which has been shown to promote prosocial behavior (Stamkou et al., 2023; O’bi and Yang, 2024). Both admiration and awe arise when seeing someone excellent with the difference that admiration likely leads to a desire to also excel at a task whereas a person experiencing awe likely feels that the excellence is beyond themselves (Keltner and Haidt, 2003; Shiota et al., 2014). In line with this reasoning, recent work by Stamkou et al. (2023) found that eliciting awe (i.e., human art) in 8- to 13-year-old children led to increased prosociality (i.e., children in the awe compared to a joy or neutral condition were more likely to donate to refugee children). Thus, seeing excellence in others, even if it is not directly related to morality, also elicits emotions that play an important role in children’s prosociality. Future research clarifying the difference between admiration and awe would be beneficial to tease apart the distinct roles such other-praising emotions play in prosocial development.

Alternatively, the lack of differences between our conditions may be because children simply enjoyed playing the Rice Game both times as it was an exciting and novel game. The Rice Game involves a tablet, a colorful screen, and food pops up in the bowl which likely elicits excitement and a feeling of being rewarded (Schacter and Jo, 2017; Papadakis et al., 2018). Some children might have been particularly excited to play the Rice Game because they have limited access to tablets and screens at home and thus, the duration of time children are engaged might be unrelated to the conditions but related to individual differences in their excitement (e.g., math ability) and exposure to screens at home (Corkin et al., 2021; Hinten et al., 2023). Previous research with adults and children implemented an arguably more ‘boring’ task such as sorting papers into different folders or math questions on a piece of paper (e.g., Schnall et al., 2010; Schnall and Roper, 2012; Stamkou et al., 2023). Thus, in future studies researchers may wish to implement a task that is less exciting for young children (e.g., boring task such as helping an experimenter to clean up the lab room) as well as a control condition (e.g., neutral social situation).

Another possibility for why we did not see differences in Rice Game engagement and accuracy between conditions might be that the stimuli used were limited in the degrees of emotion elicited. Although there were condition differences in children’s self-report of emotions that were somewhat consistent with our expectations and with prior work (i.e., Haidt, 2003; Algoe and Haidt, 2009), it is possible that the emotion experience was not strong enough to motivate behavior. The videos were 1-min long and although the experimenter presented children with a story prior to the videos, the elevation video might not have been powerful enough to elicit high prosocial motivation. Prior work with adults used a 7-min video clip (e.g., Schnall et al., 2010) and prior work with children eliciting awe used a 4-min video clip (Stamkou et al., 2023). Thus, future research might present children with longer videos or show children the same video repeatedly until they get bored. Alternatively, a live situation might be beneficial, for example, children seeing the experimenter helping someone or doing a good deed for someone during the session might elicit higher levels of elevation.

One key difference between the videos is the context. Specifically, the elevation video showed children relating to each other (i.e., interpersonal context) whereas the admiration showed individual clips of children showing their talents independently (i.e., personal context). The difference in context may be relevant when eliciting other-praising emotions and prosocial behavior as these occur in social situations. Therefore, we suggest for future research to keep the video context as similar as possible, for example, to present children with an admiration video that is also interpersonal in nature (e.g., a child practicing a handstand and seeing a friend doing a handstand in front of them).

Additionally, previous research suggests that the relation between elevation and prosocial behavior is often moderated or mediated by other variables (Diessner et al., 2013; Thomson and Siegel, 2013, 2017; Ellithorpe et al., 2015). For example, Schnall and Roper (2012) showed that only participants who engaged in a self-affirmation task (e.g., reminded themselves of previous prosocial behavior) engaged in increased prosocial behaviors after exposure to an elevation inducing stimulus. Thus, it is possible that witnessing an elevation inducing stimulus (e.g., video) alone might not be sufficient to elicit levels of elevation high enough to motivate increased prosocial behavior in children. Thus, implementing a paradigm including a self-affirmation task (e.g., experimenter asks children to recall observing prosociality in others) prior to the elevation-inducing stimulus might be useful with children. However, it is important to note that the present study was conducted with 6.5- to 8.6-year-old children, which may raise other challenges with respect to assessing children’s recall. Alternatively, future studies could use additional implicit measures to gain an index of the amount of elevation experienced by children. For example, researchers could code children’s facial expressions and body language (e.g., code levels of upliftment to indicate degree of elevation experienced) which might serve as a moderating variable.

Finally, studies suggest that there are individual differences in the degree to which individuals respond to elevation inducing stimuli (Diessner et al., 2013). Specifically, prior work shows that different personality traits are at play with different characteristics reacting differently to the environment (e.g., witnessing moral beauty) (Landis et al., 2009). Thus, we suggest for future research to look at individual differences such as personality traits as possible mediators/moderators influencing the relation between condition (elevation, admiration) and prosocial behavior.

### Gender differences in children’s baseline emotions

Our preliminary analyses revealed gender differences in children’s self-report of their emotions during the baseline phase. Specifically, girls reported higher levels of care compared to boys and boys reported higher levels of upliftment compared to girls.

Gender differences at baseline may be a result of differences in expectations such as for girls to be more caring. Such expectations are likely more pronounced in the baseline emotion phase as children were basing their baseline self-report on more general feelings (Chaplin and Aldao, 2013). Research suggests that societal expectations might involve for girls to be more relationally oriented and caring (Davis, 1995) whereas it might be more accepted for boys to be wild and energetic which might be related to upliftment. Thus, differences in emotion-self report might be due to children’s self-concepts that are linked to gender roles and expectations.

Alternatively, gender differences in the baseline phase might be due to procedural limitations. Specifically, children showed some confusion in the baseline phase as they were not sure what experience to base their reports on. For instance, it was difficult for children to respond to an emotion such as feeling upliftment when there was no ‘obvious’ reason to feel uplifted. In some instances children referred to previous experiences (e.g., “I felt uplifted when my friend gave me a present the other day”) although E explicitly asked for children to rate their emotions on the current moment. This might be the reason why we see differences in children’s baseline self-reports of upliftment between the elevation and admiration condition. Importantly however, gender differences were not found in the emotion test phase. Thus, even though the baseline measure offers some insights into the general increase or decrease of emotions after the test phase, children’s baseline reports are to be taken with caution. As mentioned above, to gain a more sophisticated understanding of children’s emotions, future research could implement implicit measures (e.g., facial expressions, body language) and physiological measures (e.g., heart rate, arousal). This could be particularly relevant for the baseline emotion phase as children were having difficulties reporting their emotions when it was not based on a stimulus.

### Extensions to the developmental literature

The current study extends prior developmental work showing that young children experience positive emotions when helping themselves (Aknin et al., 2012b; Hepach et al., 2017b) and when seeing another being helped (Hepach et al., 2012; Hepach and Tomasello, 2020) by investigating the specific emotional characteristics of seeing others being helped. Precisely, the emotional rewards associated with helping and seeing others being helped have been well documented in the literature, however, there is no study to date that has investigated whether the positive emotions experienced by young children are indeed indicators of the positive emotion of elevation. Further, there is no study to our knowledge that has examined whether the positive emotions young children experience when seeing others being helped motivates prosocial behavior. The current study revealed that the specific positive emotions children experience might indeed be indicators of elevation which is associated with feelings of happiness, warmth, and care. However, it is important to note that the current study was conducted with slightly older children (i.e., 6.5- to 8.5-years) whereas previous developmental work has been conducted with preschoolers (i.e., around 4 years) and hence, it remains unclear whether younger children also experience such nuanced emotions. One avenue for future research would be to implement the current paradigm with younger children to assess developmental change in emotion experiences.

Finally, we did not find evidence that seeing others being helped leads to increased prosocial behavior compared to seeing talent in others in 6.5 to 8.5 year old children. However, as mentioned above, this finding might be due to the contrast between elevation and admiration and thus, future work might investigate whether seeing others being helped, in contrast to a neutral social situation, results in increased prosocial behavior in preschoolers.

### Considerations and future suggestions

The current study advances the literature by introducing a novel paradigm to investigate moral emotions and prosocial behaviors in middle childhood. Even though this study offers some novel insights and important first steps shedding more light onto the role of elevation in childhood, we acknowledge that there are some limitations to consider. In line with prior work, we chose child-friendly videos from platforms such as YouTube that were engaging (e.g., Silvers and Haidt, 2008; Schnall et al., 2010 used an Oprah Winfrey Show video in which Oprah donated money). However, the videos used in the current study were not carefully designed and controlled. While we did make several attempts to reduce the impact of differences between the videos (i.e., made them the same length, adjusted the soundtracks to ensure they had instrumental music), future studies may involve original, purpose designed, videos to control for potential confounding factors (e.g., number of individuals in the video, pace, storyline, etc.).

As mentioned above, even though the Rice Game was an attractive game which most participants liked to engage with, it might not be comparable to other prosocial behavior measures examined in prior work. For instance, prior work asked adult participants to sort papers into folders or complete boring math questions on a paper (e.g., Schnall and Roper, 2012). Even though the Rice Game also involved math questions and got progressively more challenging, which we thought would not be deemed too enjoyable by children, it is a colorful game on a tablet which was likely special to children, particularly children who may have limited screen time at home. As a result, the Rice Game might be less costly to children in comparison to the prosocial behavior measures used in other studies. Moreover, the Rice Game got progressively harder, which we originally viewed as a strength as it would continue to push children’s motivation to participate. However, this feature of the game also limited our ability to standardize and control the type of questions asked. Even though our preliminary analyses looking at the number of months that children have attended school influenced our outcome measures showed no associations with our measures, children’s age was a significant predictor of accuracy (but not engagement) indicating that older children answered more questions correctly. Employing a more controlled and less exciting prosocial behavioral measure, such as volunteering to help put away toys or sort papers, may provide stronger evidence of the extent to which elevation promotes prosocial behavior in 6- to 8-year-old children.

Finally, it is important to note that there was low variability in children’s self-reported emotions. For instance, children’s baseline and test emotion self-reports were generally quite high with most children gravitating to the extremes on the circle scale [e.g., either 1 (not at all) or 6–7 (high)]. Thus, due to low variability in children’s emotion self-reports, the self-report measure might have procedural limitations to objectively assess children’s emotion experiences. As mentioned previously, supplementing the self-report measure with implicit (e.g., coding facial expressions), physiological (e.g., heart rate, arousal), or neurological (e.g., EEG and fMRI) measures likely provides a more detailed picture of children’s complex emotional experiences.

## Conclusion

Taken together, the current study builds on the adult literature by showing that elevation, in contrast to admiration, has distinct emotional characteristics. Specifically, viewing elevation-inducing content was associated with higher feelings of happiness, warmth, and care in 6.5- to 8-year-olds compared to seeing admiration-inducing content. Admiration-inducing content, in contrast, elicited higher feelings of upliftment compared to elevation-inducing content. This finding also extends the developmental literature around the positive emotions elicited when seeing others being helped by revealing that the positive emotions young children experience might indeed be signs of the positive emotion of elevation. However, our hypothesis that elevation would lead to increased prosocial behavior compared to admiration was not supported. Even though previous research has shown that inducing elevation (compared to amusement and neutral) leads to increased prosocial behavior, our findings suggest that both elevation and admiration elicit similar levels of prosociality in children. We encourage future work to implement a control variable to test whether both elevation and admiration are motivators of prosociality or whether, independent of condition, children simply enjoy playing a prosocial game.

## Data availability statement

The raw data supporting the conclusions of this article will be made available by the authors, without undue reservation.

## Ethics statement

The studies involving humans were approved by University of Auckland ethics committee. The studies were conducted in accordance with the local legislation and institutional requirements. Written informed consent for participation in this study was provided by the participants’ legal guardians/next of kin.

## Author contributions

SG: Conceptualization, Formal analysis, Investigation, Methodology, Project administration, Writing – original draft, Writing – review & editing, Data curation. RH: Conceptualization, Methodology, Supervision, Writing – review & editing. AH: Conceptualization, Data curation, Formal analysis, Funding acquisition, Methodology, Project administration, Resources, Supervision, Writing – review & editing.

## Footnotes

^1^ https://www.youtube.com/watch?v=tWXkX9axcWs

^2^ https://figshare.com/s/70c92c76d04b8934be30
