# Supplementary material for: Observing prosociality and talent: the emotional characteristics and behavioral outcomes of elevation and admiration in 6.5- to 8.5-year-old children
Source: Front Psychol. 2024 May 24;15:1392331. doi: 10.3389/fpsyg.2024.1392331 (PMC11160138; doi:10.3389/fpsyg.2024.1392331)
Supplement: Supplementary file 1 [file Table_1.DOCX]

**Supplementary Materials**

**SM1**

*Emotions used in the Baseline and Test Phases*


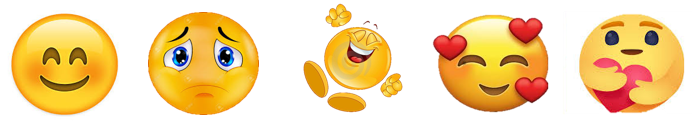


**SM2**

*Elevation Story*


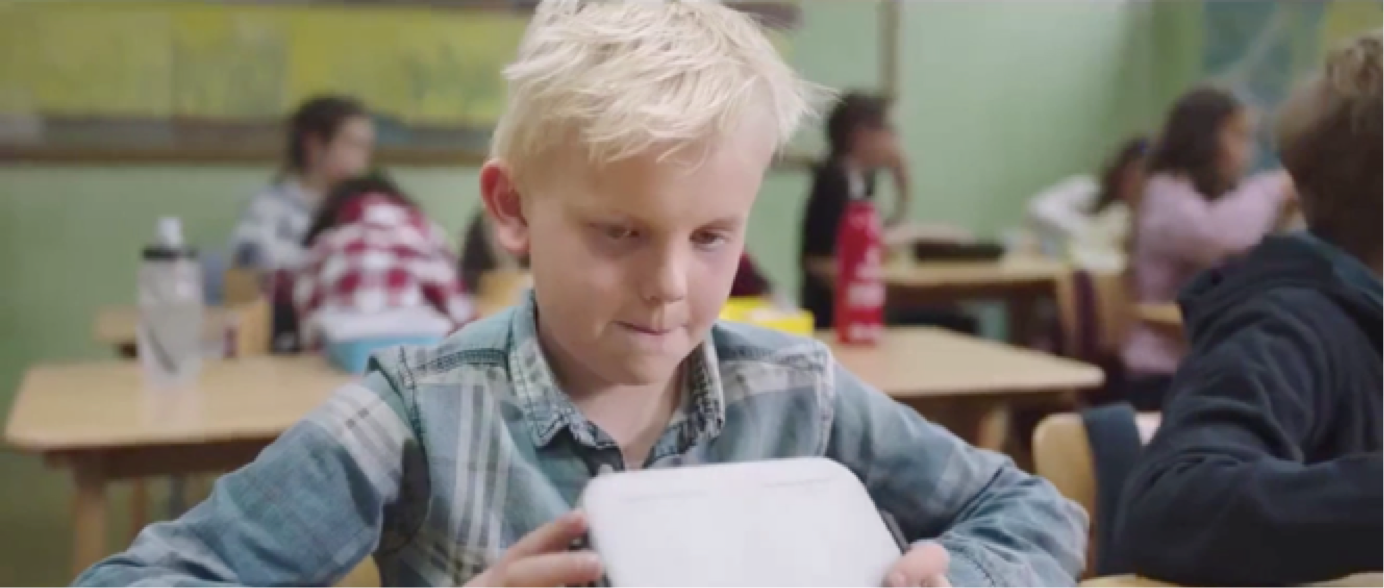

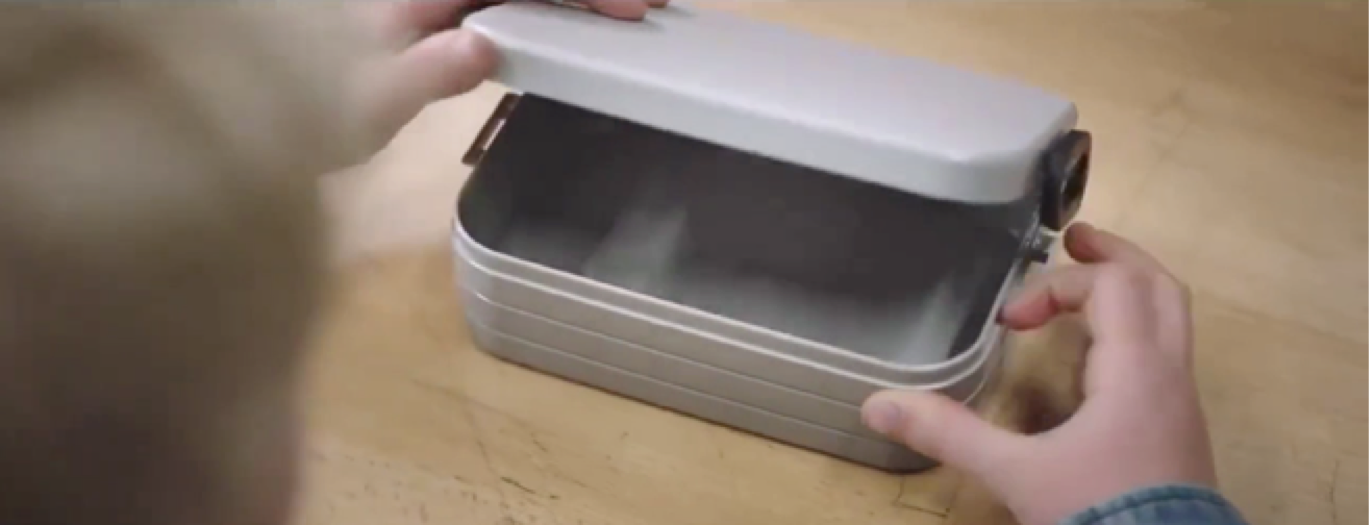

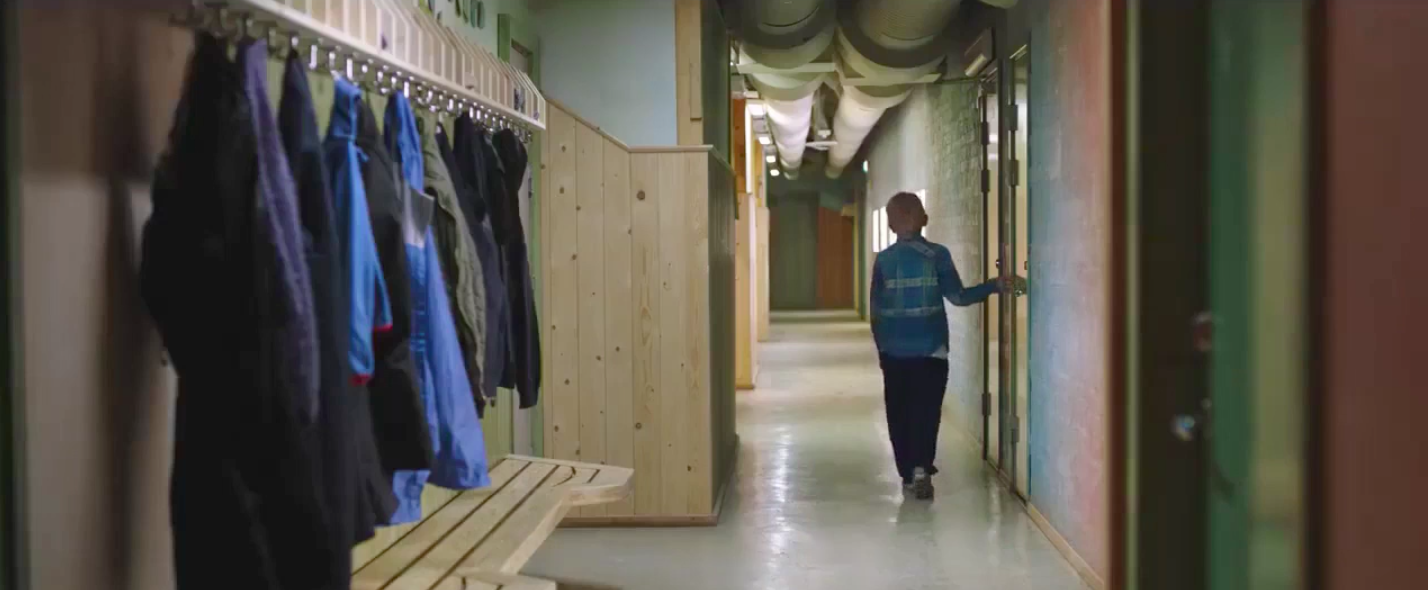


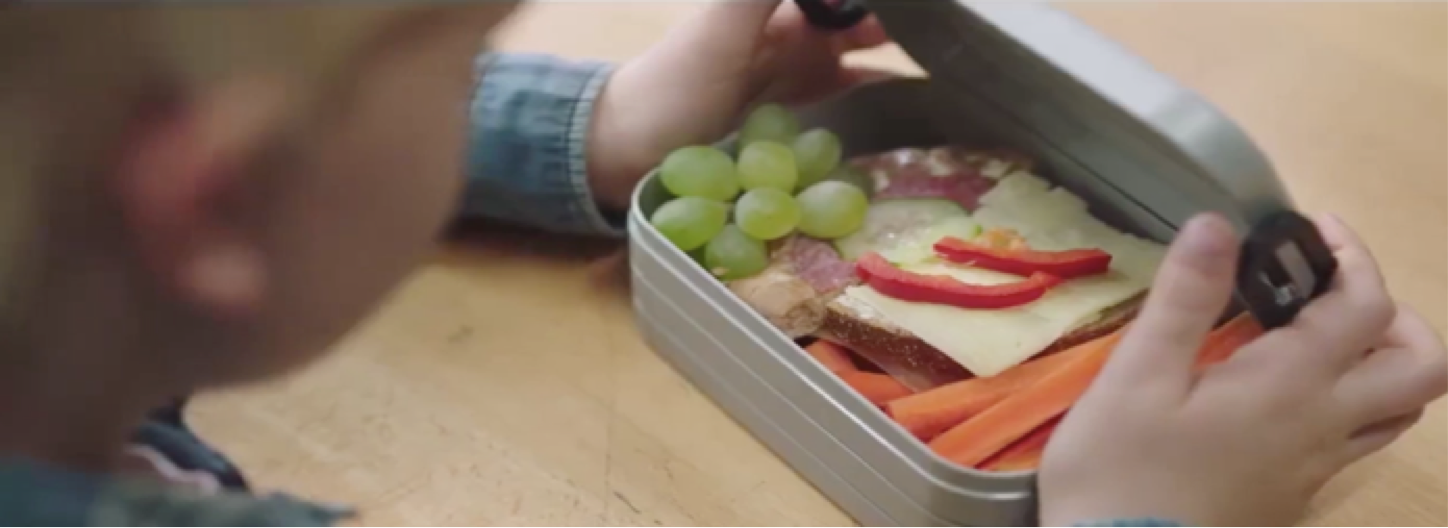

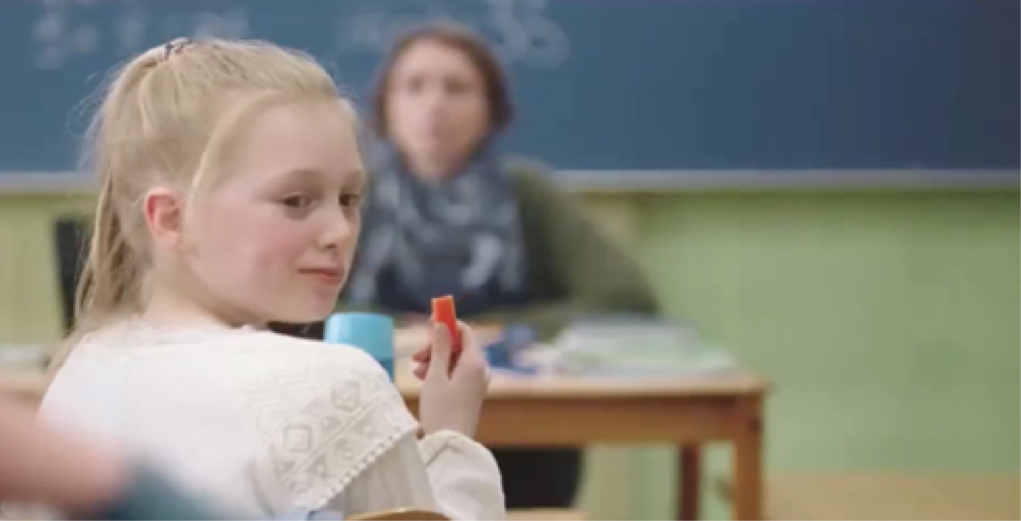

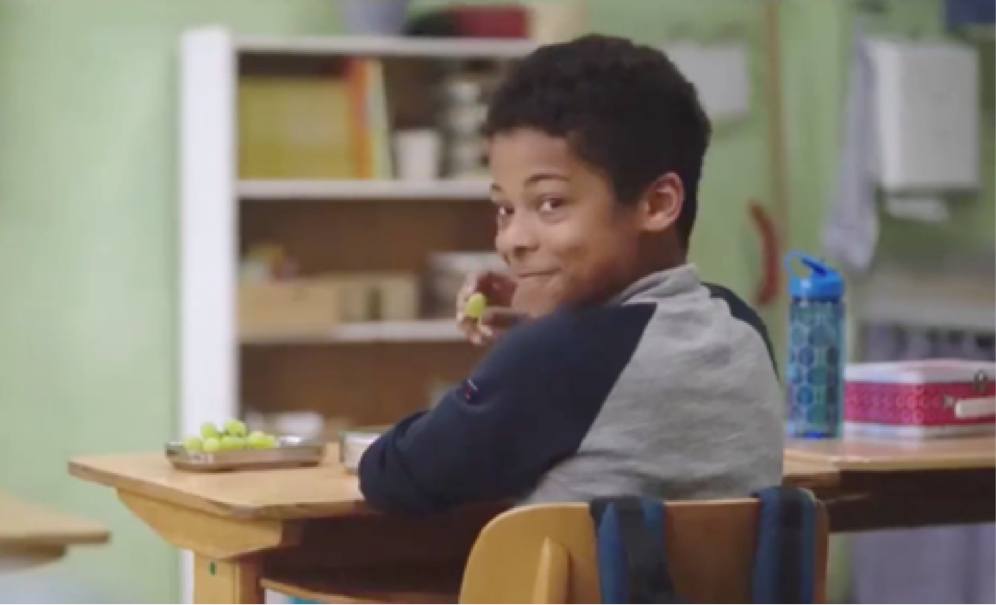

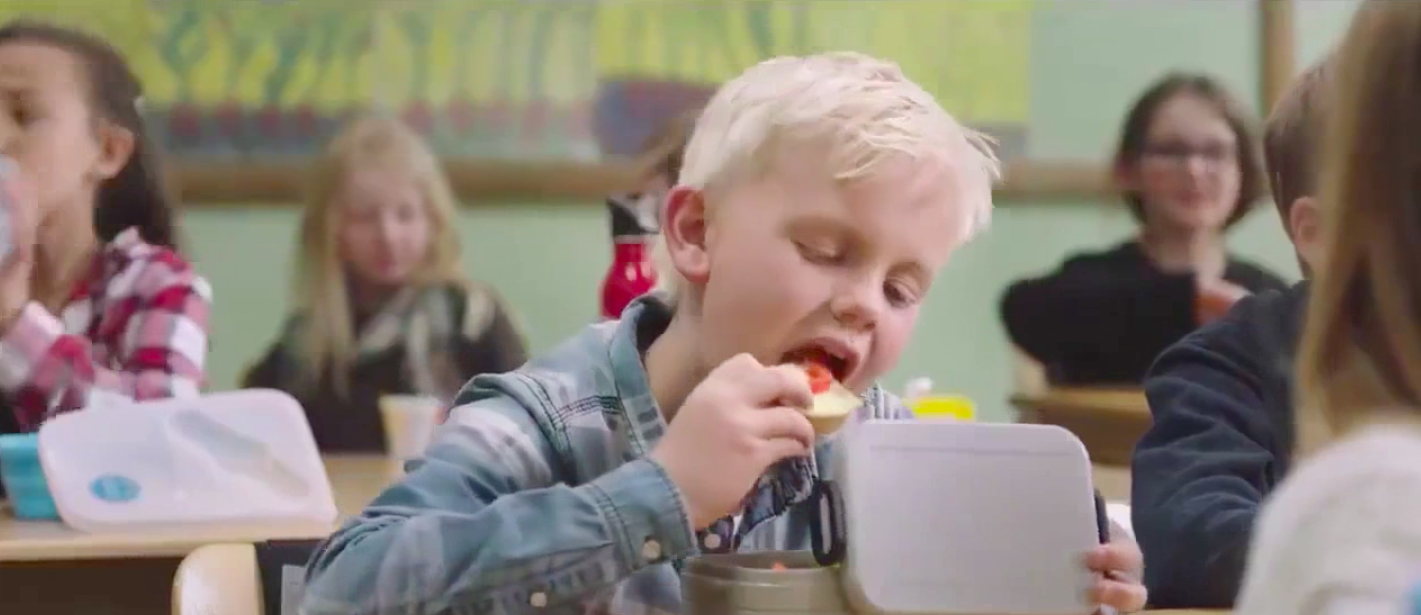


Note. Experimenter’s script: “I am going to tell you a story now. This story is about sharing and being kind to one another. Look at this boy (upper left). He is in his classroom. It is lunchtime and all the children are very hungry. All the children pick up their lunchboxes and start eating. But when this boy opens up his lunchbox it is empty (upper middle). The boy feels sad and hungry. He leaves the classroom and walks around (upper right). When he returns to the classroom he picks up his lunchbox again and now it is heavier than before. He opens it up and now there is food inside his lunchbox (bottom left). While he was outside the classroom the other children shared some of their food with the boy and put it into his lunchbox (bottom middle). The boy feels very grateful and starts eating (bottom right).

SM3

*Admiration Story*


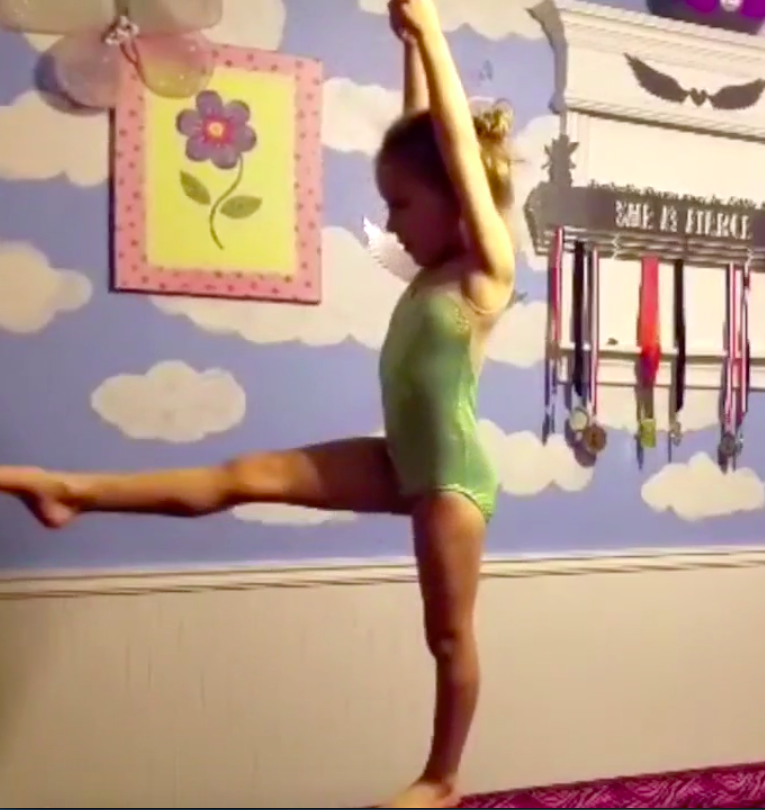

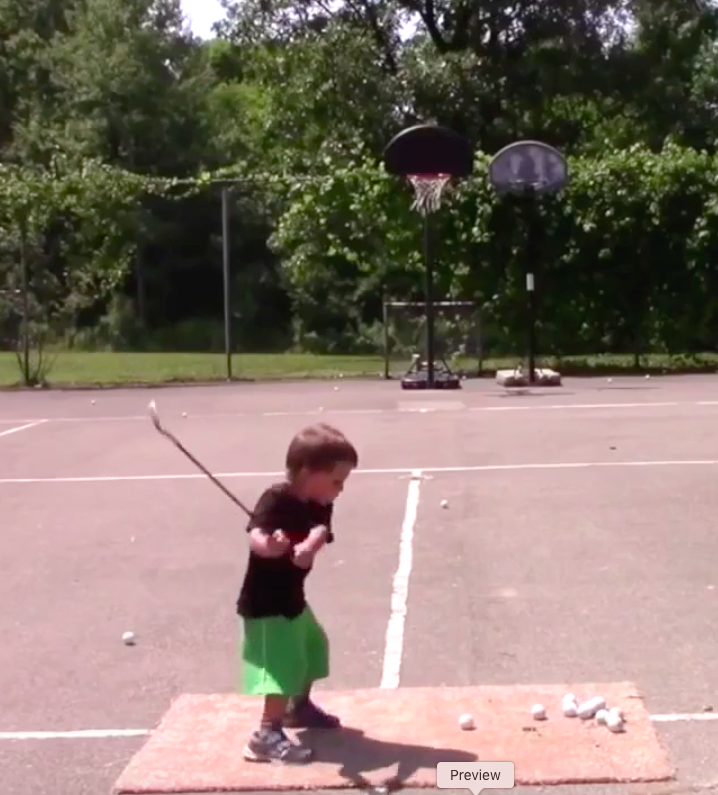

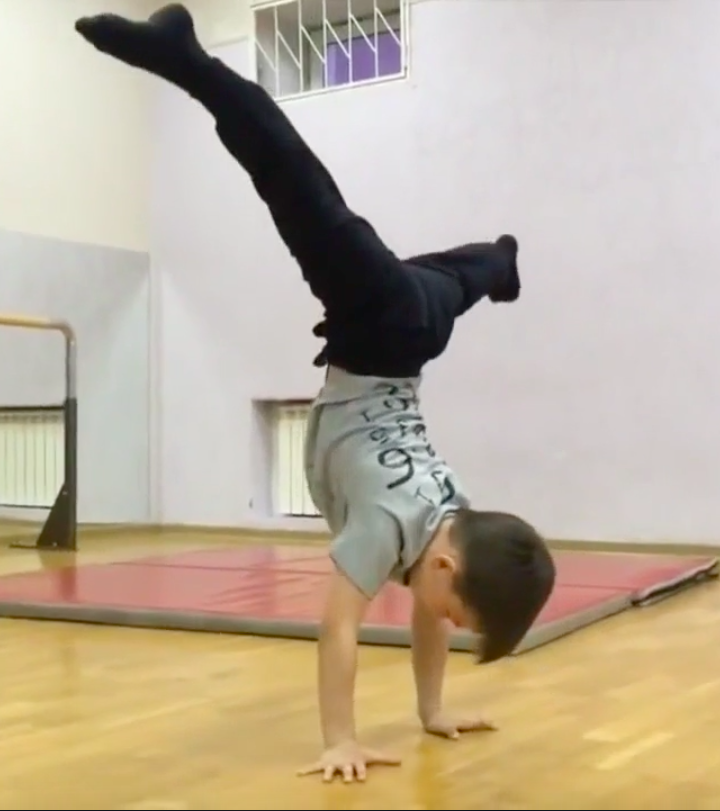

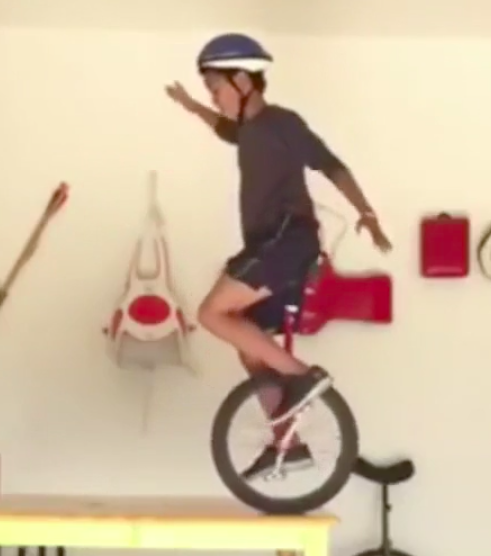


*Note.* Experimenters script: “I am going to tell you a story now. This story is about taleneted children who can do amazing things. Look at this girl (left), she does gymnastics in her room and can do flips on her bar. Look at this boy (second from left), he can hit this golf ball and goal it in the basketbal hoop which is really far away. And this boy (second from right) can do a handstand only balancing on his hans and arms. And this boy (right) can ride his unycycle on a table and he can do a back-flip on his unycycle down to the floor.”

**SM4**

Preliminary Analyses: Mask versus No-Mask

Mann-Whitney U tests revealed no differences for the emotion baseline and test phases between children who had an experimenter who wore a mask (n = 76) and did not wear a mask (n = 106): *Admiration* (Baseline (BL) happy: *Z* = -.699, *p* = .504; BL sad: *Z* = -1.83, *p* = .068; BL uplifted: *Z* = -.575, *p* = .565; BL warm and fuzzy: *Z* = -.976, *p* = .329; BL caring: *Z* = -.600, *p* = .548; Test (T) happy: *Z* = -.343, *p* = .732; T sad: *Z* = -.387, *p* = .699; T uplifted: *Z* = -.426, *p* = .670; T warm and fuzzy: *Z* = -.691, *p* = .490; T caring: *Z* = -.080, *p* = .936), *Elevation* (BL happy: *Z* = -.816, *p* = .414; BL sad: *Z* = -.865, *p* = .068; BL uplifted: *Z* = -.305, *p* = .761; BL warm and fuzzy: *Z* = -.020, *p* = .984; BL caring: *Z* = -.670, *p* = .503; T happy: *Z* = -.192, *p* = .847; T sad: *Z* = -.154, *p* = .878; T uplifted: *Z* = -.948, *p* = .343; T warm and fuzzy: *Z* = -.497, *p* = .619; T caring: *Z* = -.565, *p* = .572). Further, preliminary analyses revealed no differences for the rice game measures between experimenters who wore a mask and who did not: *Admiration* (Engagement: *Z* = -.227, *p* = .821; Accuracy: *Z* = -.993, *p* = .321), *Elevation* (Engagement: *Z* = -.398, *p* = .691; Accuracy: *Z* = -.682, *p* = .495).

**SM 5**

Preliminary Analyses: Order effects

Moreover, we tested whether there were differences in our outcome variables dependent on the order (i.e., counterbalancing, Part 1 vs Part 2) children were presented with the conditions. First, Mann Whitney U tests showed no differences for order in the admiration condition for children’s emotion self-reports: BL happy (*Z* = -.329, *p* = .742), BL sad (*Z* = -.254, *p* = .799), BL uplifted (*Z* = -.516, *p* = .606), BL warm and fuzzy (*Z* = -.602, *p* = .547), BL caring (*Z* = -.345, *p* = .730), T happy (*Z* = -.103, *p* = .918), T sad (*Z* = -1.39, *p* = .164), T uplifted (*Z* = -.951, *p* = .341), T warm and fuzzy (*Z* = -.624, *p* = .532), T caring (*Z* = -.504, *p* = .614). Second, preliminary analyses revealed a difference depending on order in the elevation condition for children’s baseline happiness with children reporting higher BL happiness scores in Part 2 compared to Part 1 (*Z* = -2.09, *p* = .037). There were no differences in order for the remaining baseline and test emotion reports in the elevation condition: BL sad (*Z* = -1.04, *p* = .300), BL uplifted (*Z* = -1.54, *p* = .123), BL warm and fuzzy (*Z* = -.948, *p* = .343), BL caring (*Z* = -.448, *p* = .654), T happy (*Z* = -.110, *p* = .912), T sad (*Z* = -.811, *p* = .418), T uplifted (*Z* = -1.22, *p* = .221), T warm and fuzzy (*Z* = -.430, *p* = .667), T caring (*Z* = -.686, *p* = .493). Finally, there were no differences for order in the rice game measures for the *Admiration* (Engagement (*Z* = -1.93, *p* = .054), Accuracy (*Z* = -1.39, *p* = .164)) and *Elevation* (Engagement (*Z* = -1.63, *p* = .104), Accuracy (*Z* = -1.38, *p* = .168) conditions.

**SM6**

Wilcoxon Signed-Rank Test results for Baseline Differences between Conditions

| *Test Statistics^a^* | | | | | |
| --- | --- | --- | --- | --- | --- |
|  | Happy | Sad | Uplifted | Warm | Caring |
| Z | -,141^b^ | -,612^b^ | -1,783^c^ | -,756^c^ | -,344^c^ |
| Asymp. Sig. (2-tailed) | ,888 | ,540 | ,075 | ,449 | ,731 |
| a. Wilcoxon Signed Ranks Test | | | | | |
| b. Based on negative ranks. | | | | | |
| c. Based on positive ranks. | | | | | |
